# Low-grade chondrosarcoma of the cricoid cartilage: a case report and review of the literature

**DOI:** 10.1007/s00256-017-2731-5

**Published:** 2017-07-29

**Authors:** Chuan-Ping Gao, Ji-Hua Liu, Feng Hou, Hua Liu, Wen-Jian Xu

**Affiliations:** 1 Department of Radiology, The Affiliated Hospital of Qingdao University, No. 16 Jiangsu Road, Qingdao, China; 2 Department of Pathology, The Affiliated Hospital of Qingdao University, No. 16 Jiangsu Road, Qingdao, China; 3 Department of Radiology, The Shinanqu People Hospital, No. 29 Guangzhou Road, Qingdao, China

**Keywords:** Tracheal tumor, Cartilaginous tumor, Chondrosarcoma, Treatment

## Abstract

We report the case of a 60-year-old man with a 12-day history of vomiting whenever he had a meal. Computed tomography revealed a calcified mass in the right cricoid cartilage with intraluminal and extraluminal extension. The patient underwent surgical resection of the trachea with end-to-end anastomosis. Pathological examination of the surgical specimen showed a low-grade chondrosarcoma. Eighteen months after surgery, the patient is alive and disease-free.

## Introduction

Primary malignant tracheal tumors are rare, accounting for only 0.2% of all malignancies of the respiratory tract. The most frequent histology is squamous cell carcinoma, followed by adenoid cystic carcinoma and cylindroma [1]. Chondrosarcoma of the trachea is a rare entity, with only 24 cases described in the literature between 1954 and 2016. In all cases, patients with a tracheal chondrosarcoma presented with variable airway obstruction. Dyspnea was the most common clinical complaint. Its nonspecific presenting symptoms often delay the diagnosis. Tumors arising from the cricoid cartilage are extremely rare. Here, we present a case of grade I tracheal chondrosarcoma. To our knowledge, it is only the second reported case of a cricoid cartilage chondrosarcoma. We also review the literature.

## Case report

**Fig. 1:**
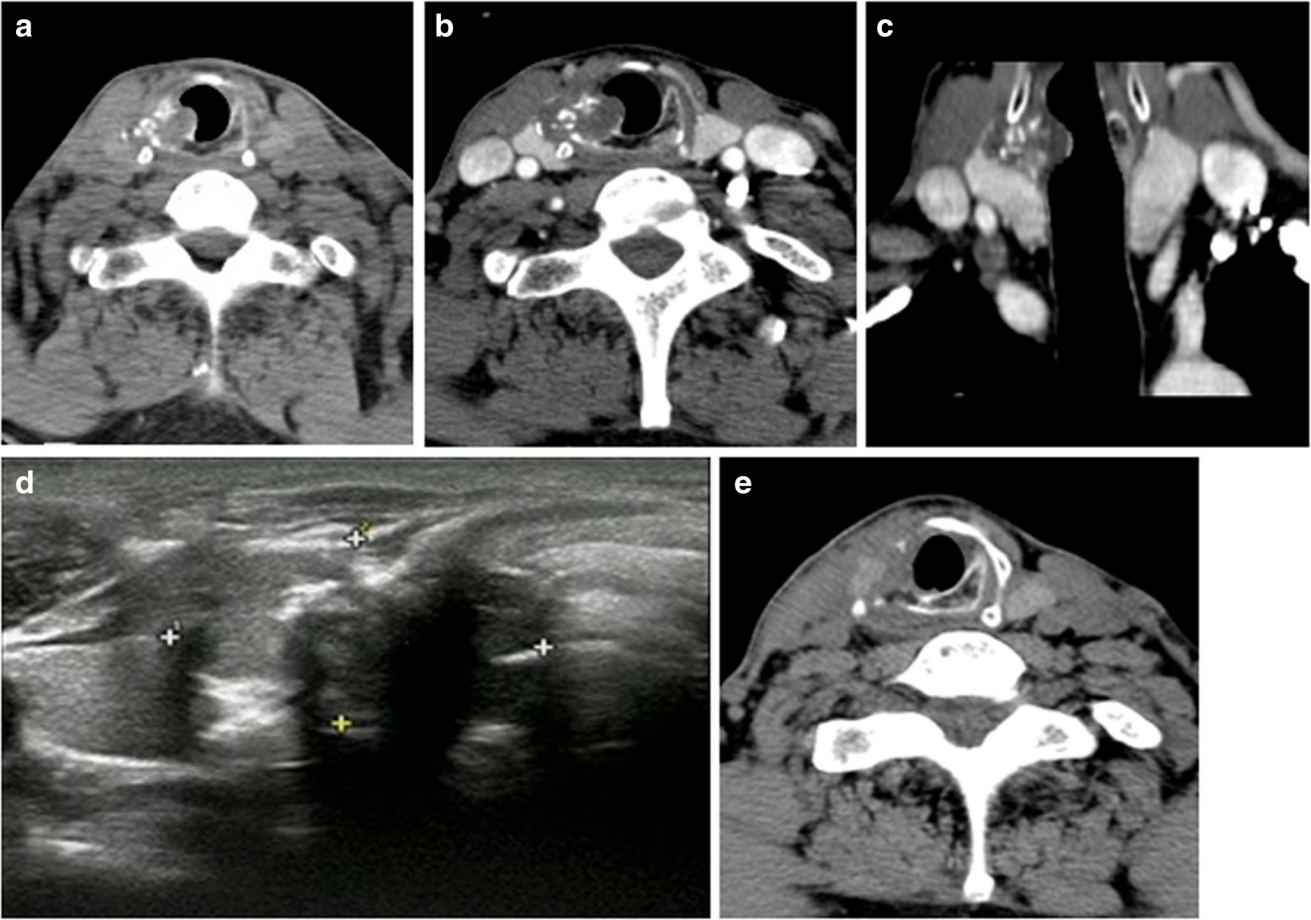
**a** Axial computed tomography shows a cricoid cartilage tumor at the conjunction of the arch and lamina on the right side. The mass contains calcifications and extends intraluminally and extraluminally. **b** There was no significant contrast enhancement. The mass compressed the right lobe of the thyroid. **c** Coronal reformatted image reveals the mass extending intraluminally and extraluminally, causing tracheal narrowing and compression of the thyroid gland. **d** Ultrasound reveals a hypoechoic mass and multiple calcifications with shadows. **e** Postoperative computed tomography shows no residual lesion

A 60-year-old man presented with a 12-day history of vomiting with each meal. On physical examination, a palpable, peanut-size, soft mass was found in the right side of his neck. There was no hoarseness. Plain radiography of the chest was normal. Computed tomography (CT) revealed an intraluminal, calcified mass on the right side, at the conjunction of the arch and lamina of the cricoid cartilage (Fig. 1a). The mass, which measured 26.3 × 20.2 × 14 mm, extended extraluminally and compressed the right lobe of the thyroid gland (Fig. 1b, c). The lesion was of heterogeneous density without significant contrast enhancement (Fig. 1b). Ultrasound revealed an irregular hypoechoic nodule adjacent to the right lobe of the thyroid gland. The lesion had destroyed the right side of the cricoid cartilage and extended intraluminally into the trachea. Multiple hyperechoic calcifications were detected in the mass (Fig. 1d). Bronchoscopy showed that nearly 20% of the lumen was obstructed. The bronchoscopic surface was relatively smooth and covered with a mucous membrane. Our preliminary diagnosis was hamartoma.

**Fig. 2:**
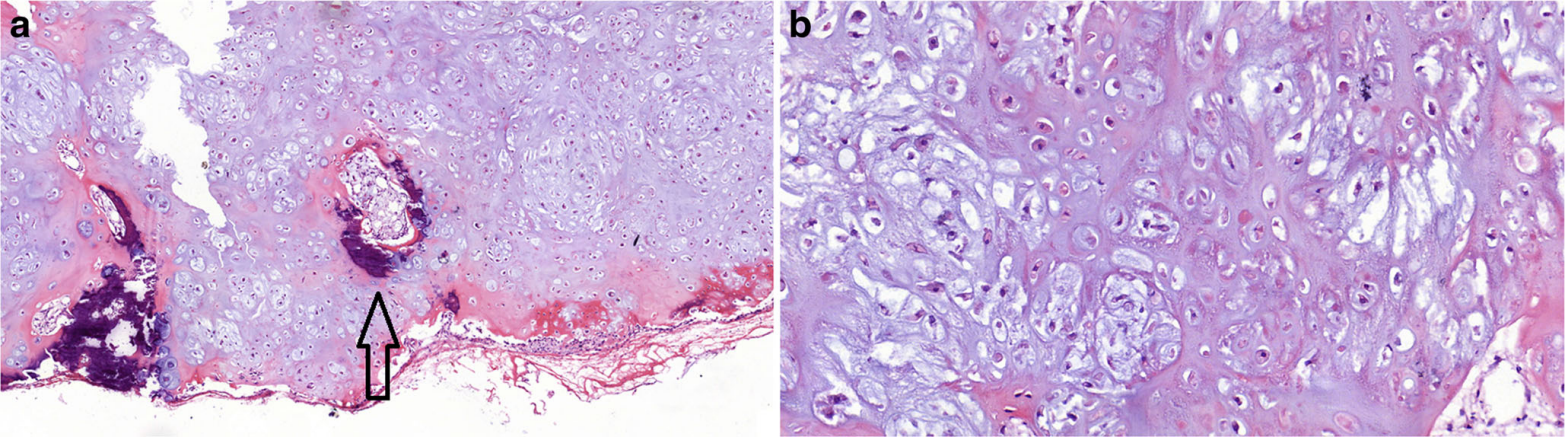
**a** Low-power microscopy shows a lobulated cartilaginous neoplasm consisting of hyaline cartilage with areas of calcification (*arrow*; H&E, ×100). **b** High-power view of the tumor shows some variation in the size and shape of the cartilage cells and binuclear cells (H&E, ×400)

The patient underwent tracheal resection. Pathological examination of the surgical specimen (Fig. 2) revealed characteristics of a low-grade chondrosarcoma and negative surgical margins (Fig. 1e). The patient was discharged on postoperative day 8 without complications.

## Discussion

"Chondrosarcoma" refers to a group of tumors that consist predominantly of cartilage. They can be classified into three histological grades (I, II, III) based on cellularity, atypia, and pleomorphism. Grade I chondrosarcoma is also called an "atypical cartilaginous tumor." Chondrosarcoma of the trachea was first described by Moersch et al. in 1954 [2]. A review of the literature revealed only 24 reported cases of tracheal chondrosarcoma. The locations of the masses ranged from the cricoid cartilage to the bronchial tree.

The etiology remains unclear, although most authors have proposed three theories: the lesions are secondary to congenital cartilaginous rests, abnormal cartilage ossification, or chondroplasia due to chronic inflammation [1, 3]. Mohajeri et al. [4] reported a tracheal chondrosarcoma in a patient with thyroid cancer who had been treated with 3,700 MBq (100 mCi) doses of radioactive iodine after thyroidectomy. Pauwels et al. [5] speculated that high doses of radioactive iodine increased the relative risk of developing certain malignancies. Malignant transformation of chondromas is uncommon. Transformation of a chondroma, however, produces a chondrosarcoma. There is one case of malignant transformation from an endotracheal chondroma in the literature. This patient was initially diagnosed with a tracheal chondroma and underwent resection. Tumor recurred with malignant transformation within 6 years of the primary operation [6].

Multiple enchondromas accompanied by multiple cutaneous hemangiomas comprise the Maffucci syndrome, which is associated with a high risk of malignant tumor development. De Almeida et al. [7] and Wagnetz et al. [8] each reported a tracheal chondrosarcoma associated with Maffucci syndrome.

Most tracheal chondrosarcomas arise from the posterolateral wall of cartilaginous rings. Twenty-four cases of chondrosarcoma have been documented in the literature since 1954 and are summarized in Table 1. Only one patient was female [26], as the lesions typically affect men 32–87 years of age. Low-grade chondrosarcoma is most common in the trachea. Among those reported, 4 of the lesions were grade II and 1 was grade III [26].

**Table 1:** Characteristics of 24 reported cases of tracheal chondrosarcoma

Reference	Age/sex	Symptoms, duration (months)	Tracheal segment involved	Calcification	Treatment	Recurrence	Follow-up (years)
Moersch et al. [2]	N/A	N/A	Tracheal bronchial tree	N/A	Resection	N/A	N/A
Jackson and Jackson [9]	32/male	C, 72	Distal trachea–right bronchus	N/A	Resection	No	6
Daniels et al. [10]	73/male	D, C, W, 8	Distal trachea—right bronchus	N/A	Resection	Local	3
Fallahnejad et al. [11]	48/female	D, C, W, 16	Proximal trachea	No	Resection	No	5
Weber et al. [12]	71/male	H, C, D, 4	Mid-distal trachea	Present	Resection	No	5
Slasky et al. [3]	58/male	D, C, 24	Distal trachea	Present	Resection	No	2.5
Arévalo et al. [13]	74/male	P, acute	2 cm below cords	Present	Resection	No	1
Matsuo et al. [14]	72/male	D, H, 7	Distal half of trachea	No	Resection	No	0.5
Mine et al. [15]	74/male	D, W, 6	Distal trachea	N/A	Resection	No	2.8
Salminen et al. [6]	57/male	P, D, 1	Distal trachea	No	Internal resection	Local + distant	14
Kaneda et al. [16]	64/male	D, 36	Distal trachea	N/A	Resection	No	1
Leach et al. [17]	78/male	C, 36	Distal trachea	Present	Resection	No	N/A
Kiriyama et al. [18]	54/male	W, D, 1	Distal trachea	Present	Resection	No	3.5
Hervás et al. [19]	84/male	C, D, St, 24	Middle trachea	N/A	Resection	No	3
Farrell et al. [20]	87/male	D, 12	4 cm below cords	N/A	Radiotherapy	Local	1
Aznar et al. [21]	49/male	St, D, acute	Proximal trachea	N/A	Resection	No	4
Maish and Vaporciyan [1]	78/male	D, 3	Distal trachea	No	Resection	No	0.5
Umezu et al. [22]	34/male	H, D, 8	Proximal trachea	Present	Resection	No	6
Wagnetz et al. [8]	34/male	C, W, D, 18	2 cm below cricoid	Present	Resection	No	N/A
Mendonça et al. [23]	72/male	O, D, W, 12	Proximal trachea	Present	Resection + radiotherapy	No	7
De Almeida et al.[7]	35/male	D, W	Proximal trachea	Present	Resection	No	N/A
Mirza et al. [24]	63/male	W, D, 6	Middle trachea	N/A	Resection	No	0.8
Mohajeri et al. [4]	74/male	D, H, St	Proximal trachea	Present	Resection	No	N/A
Andolfi et al. [25]	79/male	W, 36	Proximal trachea	Present	Resection	No	0.75

*C* cough,* D* dyspnea,* W* wheezing,* H* hemoptysis,* P* pneumonia,* St* stridor, *O* odynophagia

Grossly, tracheal chondrosarcoma is well circumscribed and commonly covered with normal mucosa. It has been uniformly described as a firm, gray–white, multi-lobulated mass with multiple calcifications. Histopathologically, the cartilaginous tissue is usually hypercellular. Low-grade chondrosarcoma contains round and polygonal cells, nuclear hyperchromasia, large nucleolated nuclei with open chromatin, and occasionally giant nuclei [22, 27]. Low-grade chondrosarcoma resembles benign chondroma, and it is difficult to differentiate the two lesions based on their histological features alone. However, different kinds of calcifications are present [1, 22]. The intermediate-grade neoplasm has irregular, ill-defined lobules. The nuclei exhibit moderate to focally marked pleomorphism, and many lacunae contained more than one cell [20].

The tracheal chondrosarcoma may cause an obstructive syndrome depending on whether the tumor grows intraluminally or extends through the tracheal wall. The common symptoms are chronic nonproductive cough, dyspnea, and stridor, which are thought to occur when more than 75% of the tracheal lumen is occluded. Unlike previously reported cases, the patient presented herein, although vomiting after meals, showed no obstructive symptoms because the tumor had a small intraluminal component. A patient with a tracheal lesion who presents with vomiting is rather unusual, so we speculated that the vomiting was caused by the mass compressing and irritating the recurrent laryngeal nerve.

Computed tomography (CT) is the modality of choice for evaluating the characteristics of tracheal tumors. It allows evaluation of the location, size, calcification, degree of obstruction, and extra-tracheal extension. Tumors occurring in the trachea and arising from the cricoid and bronchial tree are rare. Aznar et al. [21] described the first case of primary cricoid cartilage chondrosarcoma in 2001. We report the second documented case. The tumor in the present case arose from the conjunction of the arch and lamina of the cricoid cartilage and contained multiple calcifications. The mass showed no significant contrast enhancement, but did extend intraluminally and extraluminally, causing tracheal narrowing and compression of the right lobe of the thyroid gland. The tumor was classified as stage IA according to the TNM staging system because there was no lymphadenopathy.

Chondrosarcomas range in diameter from 2.0 to 6.5 cm [22]. Calcification is seen in 75% of these cartilaginous tumors and may be punctate or linear and central or peripheral [12]. We have found that tracheal chondrosarcomas frequently present with calcification (detected in 12 of the 25 cases), which has also been identified in tracheal hamartomas. In hamartomas, however, the calcification is usually limited by the tracheal cartilage and so is restricted to the tracheal lumen. Areas of decreased attenuation in the mass had CT numbers compatible with those of fat, which can be confidently used to diagnose the lesion as a hamartoma [28]. Chondrosarcomas are more likely to cause thickening of the tracheal wall and to exhibit extraluminal extension, causing tracheal narrowing. Two cases of well-differentiated chondrosarcoma were seen with contrast-enhanced CT. On the other hand, there was no significant contrast enhancement in the case reported by Andolfi et al. [25] or in the present case. Magnetic resonance imaging has no added value compared with CT and may overlook calcifications. Ultrasound may be helpful for establishing the relationship between the mass and the thyroid gland. Calcification appears as a high echo with a sound shadow behind it on ultrasound, which is detected in chondrosarcomas and in hamartomas.

The optimal treatment of tracheal chondrosarcoma is tracheal resection with free margins and end-to-end anastomosis [20]. Among the 25 known cases, 22 underwent complete resection (including the present case), with an excellent prognosis in 18 of the 22 cases. There was no local recurrence or remote metastasis after 6 months to 7 years of follow-up. One patient underwent incomplete resection for a chondroma, with malignant transformation after 6 years. The patient died 3 years after the tumor had been verified as malignant [6]. Chondrosarcoma, especially of lower grade, seems to be unresponsive to chemotherapy. Farrell et al. [20] reported one patient with an intermediate-grade (grade 2 on a 1–3 scale) tracheal chondrosarcoma who refused surgical resection and underwent radiotherapy (4,000 cGy). The lesion did not increase in size, but the patient was observed to suffer severe dyspnea at the 12-month follow-up.

In conclusion, tracheal chondrosarcoma remains a rare but surgically treatable tumor. Generally, this tumor arises from the tracheal cartilaginous ring and displays intraluminal and extraluminal extension, causing tracheal narrowing. CT and ultrasound are sensitive tools for detecting calcifications scattered in these lesions in a variety of patterns. The tumor is a relatively slow-growing lesion. Its nonspecific symptoms may delay diagnosis. The treatment of choice is tracheal resection with free margins and end-to-end anastomosis. Tracheal chondrosarcoma appears to behave in a benign fashion if adequate resection has occurred. Incomplete resection is complicated by a high local recurrence rate. For patients whose disease recurs or is deemed unresectable, radiotherapy may provide local control.
